# Update on the Status of *Bemisia tabaci* in the UK and the Use of Entomopathogenic Fungi within Eradication Programmes

**DOI:** 10.3390/insects4020198

**Published:** 2013-05-16

**Authors:** Andrew G. S. Cuthbertson

**Affiliations:** The Food and Environment Research Agency, Sand Hutton, York YO41 1LZ, UK; E-Mail: andrew.cuthbertson@fera.gsi.gov.uk; Tel.: +44-0-1904-462201; Fax: +44-0-1904-462111

**Keywords:** *Beauveria bassiana*, *Bemisia tabaci*, biological control, *Lecanicillium muscarium*

## Abstract

The sweetpotato whitefly *Bemisia tabaci* (Gennadius) (Hemiptera: Aleyrodidae) continues to be a serious threat to crops worldwide. The UK holds Protected Zone status against this pest and, as a result, *B. tabaci* entering on plant material is subjected to a policy of eradication. Both B and Q *Bemisia* biotypes are now regularly intercepted entering the UK. With increasing reports of neonicotinoid resistance in both these biotypes, it is becoming more problematic to control/eradicate. Therefore, alternative means of control are necessary. Entomopathogenic fungi (*Lecanicilllium muscarium* and *Beauveria bassiana*) offer much potential as control agents of *B. tabaci* within eradication programmes in the UK.

## 1. Introduction

**Figure 1:**
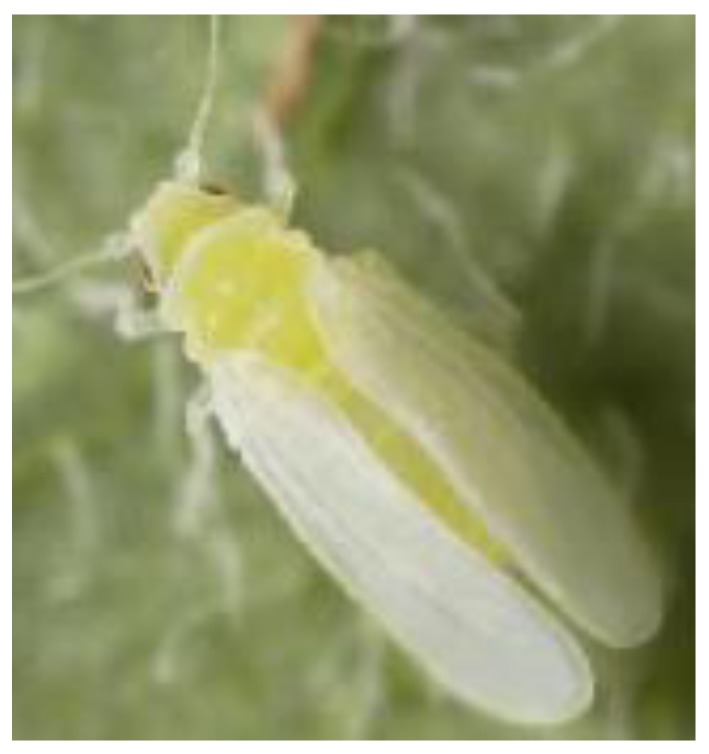
The sweetpotato whitefly, *Bemisia tabaci* (UK Crown Copyright©).

The sweetpotato whitefly, *Bemisia tabaci* Gennadius (Hemiptera: Aleyrodidae) (Figure 1) is a major pest of economically important crops worldwide [1]. *Bemisia tabaci* damages crops by feeding on phloem sap and the large amounts of sticky honeydew produced can lower the rate of leaf photosynthesis. This whitefly is also a vector of many plant viruses [2]. Within the United Kingdom (UK) (a designated Protected Zone), *B. tabaci* remains a notifiable pest subject to a policy of eradication if found on propagators premises, plants moving in trade, and containment/eradication if outbreaks occur at nurseries [3,4]. *Bemisia tabaci* has been intercepted annually on imported plant material since 1987 [4]. The primary concern is that the whitefly imported on ornamental plants such as poinsettia (*Euphorbia pulcherrima*) can transfer and infect tomatoes with Tomato yellow leaf curl virus (TYLCV) and Tomato yellow leaf curl Sardinia virus (TYLCSV) both of which are not currently present in the UK. Since the review of Cuthbertson *et al*. [4], research has shown a gradual shift from *Bemisia tabaci* B (Middle East-Asia Minor 1 species) to *Bemisia tabaci* Q (Mediterranean species) biotype being regularly intercepted on plant material entering the UK [2,5,6]. 

## 2. Pest Status of *Bemisia tabaci*

The pest status of *B. tabaci* insects is complicated by the recognition of 11 well-defined genetic groups and at least 24 morphocryptic species which are morphologically identical but distinguishable at the molecular level [7,8]. Formerly the term biotypes was used to define and discriminate *B. tabaci* populations with very different biological characteristics including invasiveness, insecticide resistance profile, vector competence and host ranges [9]. It is the B biotype (Middle East-Asia Minor 1 species) and Q biotype (Mediterranean species) that are the most invasive and damaging *B. tabaci* biotypes around the world, presenting the greatest threat to glasshouse crops [10]. The damaging B biotype is an aggressive coloniser and it is an effective vector of viruses, whereas the Q biotype characteristically shows strong resistance to novel insecticides [11,12]. There are several active ingredients currently used in the UK for treating *B. tabaci* outbreaks [4,13], but with increasing chemical resistance being shown by *B. tabaci* [14,15,16,17,18] an integrated strategy using both biological and chemical agents is required [19].

## 3. UK Interceptions of *Bemisia tabaci*

**Figure 2:**
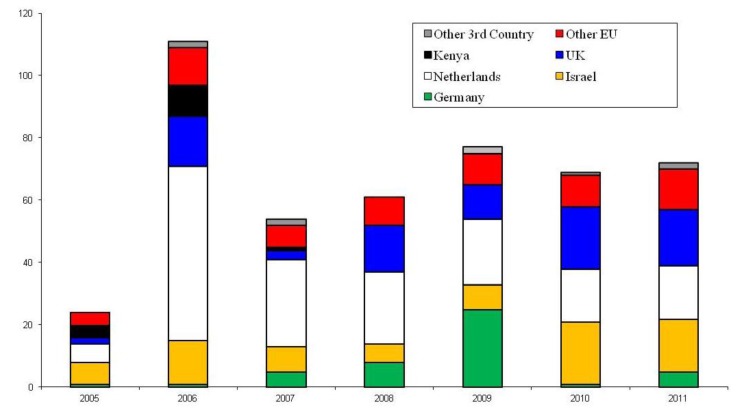
Number and source of *Bemisia tabaci* interceptions at growing sites in the UK (2005-11). Data source: The Food and Environment Research Agency, York, UK.

**Figure 3:**
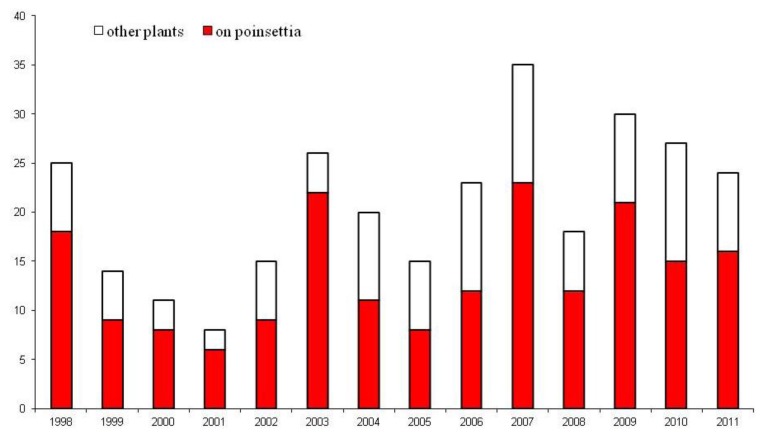
The number of *Bemisia tabaci* outbreaks in England and Wales (1998–2011). Data source: The Food and Environment Research Agency, York, UK.

Since 1987, *B. tabaci* has been intercepted at growing sites on an extremely wide range of hosts at nurseries. Interceptions of *B. tabaci* coming into the UK would appear to follow no pattern with numbers of interceptions from source countries varying from year to year (Figure 2). The majority of *B. tabaci* outbreaks within England and Wales are on poinsettia plants (Figure 3). In the effort to maintain the UK’s Protected Zone status against this global pest, much research has been undertaken at The Food and Environment Research Agency (FERA) in York on the development of eradication strategies against *B. tabaci*. The work has concentrated heavily on the use of entomopathogenic fungi, namely, *Lecanicillium muscarium* and *Beauveria bassiana*.

## 4. Entomopathogenic Fungi for the Control of *Bemisia tabaci*

**Figure 4:**
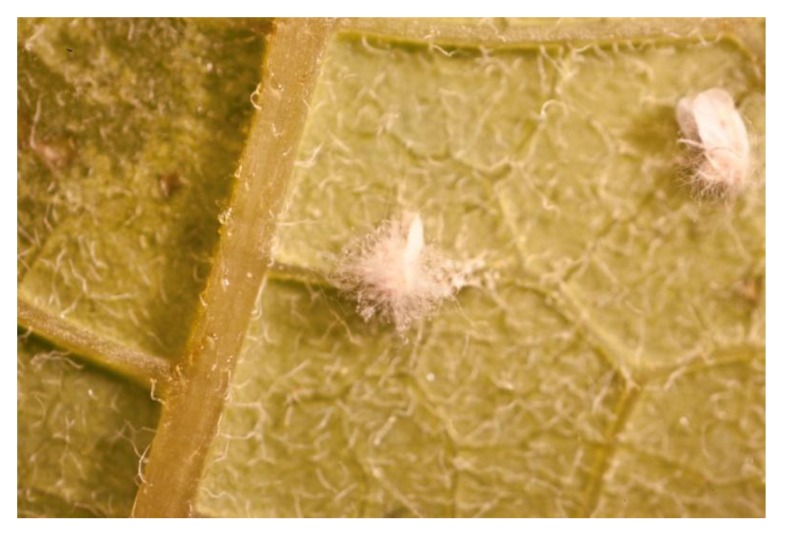
*Bemisia tabaci* adults infested with *Beauveria bassiana* (UK Crown copyright©).

Several entomopathogenic fungi have been recognized as important biological control agents of aleyrodid pests [20,21,22]. More than 20 species of entomopathogenic fungi are known to infect whiteflies [20,23,24], but *Paecilomyces fumosoroseus* and *Lecanicillium muscarium* have been most widely studied [25]. *Beauveria bassiana* also readily infects adult *Bemisia tabaci* [19] (Figure 4).

**Figure 5:**
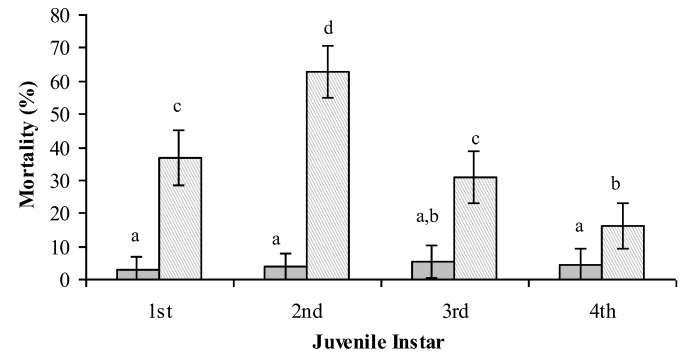
The susceptibility of the immature stages of *Bemisia tabaci* to the entomopathogenic fungus *Lecanicillium muscarium* on verbena plants.Columns with the same letter are not significantly different. Water control; 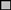 *Lecanicillium muscarium* + 0.02% Agral 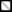 [26].

A study by Cuthbertson *et al*. [26] confirmed that second instar *B. tabaci* were generally the most susceptible life stage to infection by *L. muscarium* on tomato and verbena infected foliage (Figure 5). The results obtained are similar to those recorded for this fungus against *B. tabaci* on poinsettia plants [27].

Due to the differences in sensitivity of fungal species to different formulations of the same insecticide, information is required on the compatibility of each entomopathogenic fungi and chemical product to be used within a given control/eradication strategy. Formulations of different insecticides may differ in toxicity to fungi due to the use of different surfactants. Further, fungi species may also differ in sensitivity to different formulations of the same insecticide. Therefore, information regarding compatibility between entomopathogenic fungi and each chemical product for a control programme needs to be tested individually within the ecosystem in which it will be applied. 

Within the UK, only Cuthbertson *et al.* [19,28,29] have investigated the combination of chemicals routinely used for the control of whitefly with fungi. Here varying results have been obtained. In regards to mixing chemicals with *L. muscarium*, direct exposure for 24 hours to imidacloprid, nicotine and teflubenzuron resulted in very low spore germination, unsuitable for commercial use. Only the active ingredient buprofezin provided an acceptable level of spore germination [28].

**Figure 6:**
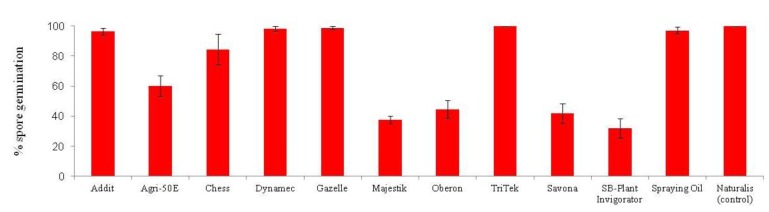
Effects of direct tank mixing of *Beauveria bassiana* with a range of chemical products [19].

The implementation of a control programme may require sequential rather than simultaneous applications of insecticides and entomopathogenic fungi but few previous studies have tested the effect of dry insecticide residues on fungal activity. Recent work [28] has shown that when *L. muscarium* was applied to plants sprayed 24 hours earlier with a standard commercial application of one of three contact insecticides or with treatment using a systemic insecticide, no significant reduction in infectivity (mycelial growth) was detected in any cases. Therefore, *L. muscarium* could be applied sequentially with imidacloprid, buprofezin, nicotine and teflubenzuron in a commercial control strategy. In similar trials, *B. bassiana* proved suitable for direct tank mixing with a range of products including petroleum oils [19] (Figure 6) with no detrimental effect on spore germination. Following sequential applications of *L. muscarium* and chemicals, mortalities of up to 90% of *B. tabaci* second instars were recorded [28]. Sequential treatments offer a greater flexibility in timing applications against various life stages of the pest. 

The chemical groups most toxic to fungi are organophosphates and carbamates. Many commonly used insecticides, for example, buprofezin, have now been rendered ineffective in the UK against *Trialeurodes vaporariorum* (glasshouse whitefly) by the widespread appearance of resistance in populations. This product has now also just recently become unavailable for use in UK horticulture [4]. *Bemisia tabaci* have also been shown to offer a degree of resistance to imidacloprid [17] adding urgency to the development of alternative control approaches. 

The ambient temperature and humidity are known to be important factors determining fungi efficacy. Trials have shown that for optimal use of *L. muscarium* and *B. bassiana* favourable conditions for fungi survival and efficacy must be maintained for up to 6–8 hours following application to plant foliage [19,30].

## 5. Conclusions

*Lecanicillium muscarium* and *B. bassiana* have the potential to be important components of eradication strategies for use in the UK against *B. tabaci* [19,30]. Integrated approaches utilizing entomopathogens are showing much potential. Both early instars and adults of *B. tabaci* are proving very susceptible to infection. The levels of both direct and indirect compatibility of the fungi with chemical insecticides also increase their potential for incorporating them into strategies for the eradication of *B. tabaci*. Their use now depends on further work in commercial-scale glasshouse crops and, if successful, they may contribute to the development of sustainable production systems through a reduction in the use of chemical insecticides and, consequently, a reduction of chemical residues on produce and insecticide resistance. Further research is now required to both fine tune the application techniques and optimum dose rates required for their use within the glasshouse environment.
